# For control of COVID-19: the development of regular mass testing of COVID-19 in old age psychiatry wards

**DOI:** 10.1192/j.eurpsy.2021.688

**Published:** 2021-08-13

**Authors:** J. Baek, S. Lennon

**Affiliations:** 1 Old Age Psychiatry, Greater Manchester Mental Health NHS FT, LT, United Kingdom; 2 Old Age Psychiatry, Greater Manchester Mental Health NHS FT, Manchester, United Kingdom

**Keywords:** COVID-19, Mass testing, old age psychiatry, SARS-CoV-2

## Abstract

**Introduction:**

COVID-19, was declared a pandemic by World Health Organisation on March 11, 2020. Older people with dementia or those with multimorbidity are more vulnerable to infection with severe acute respiratory syndrome coronavirus 2 (SARS-CoV-2), the virus responsible for the development of COVID-19. Given absence of a vaccine or treatment, prevention is the fundamental aspect of COVID-19 control. This requires early identification of contagious people with COVID-19 and isolation keeping them apart from non infected group of people. Early identification of infection in elderly with dementia or functional psychiatric condition is often difficult, due to difficulty in obtaining history or evaluating medical symptoms.

**Objectives:**

1) To establish the current standards of interventions provided at the unit to control COVID-19, with current recommendation by Government guidance. 2) To address difficulties in early identification of people of COVID-19 in Old Age Psychiatry wards. 3) To introduce sustainable interventions aimed at controlling COVID-19 risk, targeted to this group.

**Methods:**

Trust guidance for COVID-19 testing on the ward and guidance of isolation were reviewed. Literature review of currently available scientific evidence for testing for controlling COVID-19 was conducted.

**Results:**

We have created a bi-weekly mass testing guidance for Old Age Psychiatry inpatient wards with clear guidane of when to start isolation and when to stop isolation.

**Conclusions:**

There is no specific interventions to target older adult within our service currently and it was felt that it is necessary to develop a sustainable mass testing programme for this group of people for control of COVID-19.

